# Antiparasite treatments reduce humoral immunity and impact oxidative status in raptor nestlings

**DOI:** 10.1002/ece3.891

**Published:** 2013-11-22

**Authors:** Sveinn Are Hanssen, Jan Ove Bustnes, Lisbeth Schnug, Sophie Bourgeon, Trond Vidar Johnsen, Manuel Ballesteros, Christian Sonne, Dorte Herzke, Igor Eulaers, Veerle L B Jaspers, Adrian Covaci, Marcel Eens, Duncan J Halley, Truls Moum, Rolf Anker Ims, Kjell Einar Erikstad

**Affiliations:** 1 Norwegian Institute for Nature Research, Unit for Arctic Ecology, Fram CentreN-9296, Tromsø, Norway; 2 Norwegian Institute for Agricultural and Environmental Research, Soil, Water and Environment DivisionFr. A. Dahlsvei 20, N-1432, Ås, Norway; 3 Faculty of Science and Technology, Department of Bioscience, Aarhus UniversityFrederiksborgvej 399, PO Box 358, DK-4000, Roskilde, Denmark; 4 Norwegian Institute for Air Research, Fram CentreN-9296, Tromsø, Norway; 5 Ethology Research Group, Department of Biology and Toxicological Centre, University of AntwerpUniversiteitsplein 1, B-2610, Wilrijk, Belgium; 6 Department of Biology, Norwegian University of Science and Technology (NTNU)7491, Trondheim, Norway; 7 Department of Terrestrial Ecology, Norwegian Institute for Nature ResearchTungasletta 2, N-7485, Trondheim, Norway; 8 Faculty of Biosciences and Aquaculture, Marine Genomics group, University of NordlandN-8049, Bodø, Norway

**Keywords:** Costs of parasitism, immunoecology, immunoglobulin, oxidative status.

## Abstract

Parasites are natural stressors that may have multiple negative effects on their host as they usurp energy and nutrients and may lead to costly immune responses that may cause oxidative stress. At early stages, animals may be more sensitive to infectious organisms because of their rapid growth and partly immature immune system. The objective of this study was to explore effects of parasites by treating chicks of two raptor species (northern goshawk *Accipiter gentilis* and white-tailed sea eagle *Haliaeetus albicilla*) against both endoparasites (internal parasites) and ectoparasites (external parasites). Nests were either treated against ectoparasites by spraying with pyrethrin or left unsprayed as control nests. Within each nest, chicks were randomly orally treated with either an antihelminthic medication (fenbendazole) or sterile water as control treatment. We investigated treatment effects on plasma (1) total antioxidant capacity TAC (an index of nonenzymatic circulating antioxidant defenses), (2) total oxidant status TOS (a measure of plasmatic oxidants), and (3) immunoglobulin levels (a measure of humoral immune function). Treatment against ectoparasites led to a reduction in circulating immunoglobulin plasma levels in male chicks. TOS was higher when not receiving any parasite reduction treatment and when receiving both endo- and ectoparasitic reduction treatment compared with receiving only one treatment. TAC was higher in all treatment groups, when compared to controls. Despite the relatively low sample size, this experimental study suggests complex but similar relationships between treatment groups and oxidative status and immunoglobulin levels in two raptor species.

## Introduction

Natural stressors such as infectious organisms are potentially costly to wild organisms, as they use their hosts' resources for own survival and reproduction, and because the hosts' immune defenses may be energy demanding (de Lope et al. 1998). In addition, immunological activity, especially the inflammatory response (Sorci and Faivre 2009), and metabolic processes lead to the production of reactive oxygen species (ROS) (Halliwell and Gutteridge 2007) that may have serious cytotoxic effects for the hosts as they are able to oxidize macromolecules such as proteins or DNA (Finkel and Holbrook 2000; Fang et al. 2002). Oxidative damage is also suggested to be one of the key physiological mechanisms of the aging process (Barja 2000, 2004; but see Kregel and Zhang 2007; Buttemer et al. 2010). Both endogenous (e.g., superoxide dismutase) and dietary antioxidants (e.g., vitamins E and C) are able to break down ROS and offer protection against oxidative damage (Finkel and Holbrook 2000; Barja 2004). It should be noted here that some birds are also able to synthesize vitamin C so it might not be entirely dietary in raptors (e.g., Chaudhuri and Chatterjee 1969). As ROS production exceeds the antioxidant capacity, the damaging effects of ROS will materialize (Balaban et al. 2005; Costantini 2008; Monaghan et al. 2009). Oxidative stress may appear as a disturbance in the pro-oxidant/antioxidant balance in favor of the oxidants, leading to a disruption of redox signaling and control and/or molecular damage (Sies and Jones 2007), and may impair viability and other fitness-related components (Beckman and Ames 1998; Noguera et al. 2012).

Infectious organisms may be more detrimental at early developmental stages when rapid growth implies a high energy and nutrient demand and given that the immune system is then still partly immature (Janeway et al. 1999). During postnatal development, both the intense metabolism and immune responses may cause massive ROS production (Halliwell and Gutteridge 2007; Martin and Schwabl 2008; Nussey et al. 2009). In addition, sex differences in androgen (e.g., testosterone) levels may lead to intersex differences in immune function and thus parasite resistance (Gause and Marsh 1986; Folstad and Karter 1992; Schuurs et al. 1992). Furthermore, sexual size dimorphism may also cause faster and thus more energy demanding growth in the larger sex (Owens and Hartley 1998).

Parasites may be classified as either endoparasites (internal parasites) or ectoparasites (external parasites). Many of the large endoparasites are located in the digestive system of their host where they passively absorb energy, often attaching to their hosts intestines by various hooks or spikes causing local lesions and inflammation (Schmid-Hempel 2011). Ectoparasites, on the other hand, are often arthropods that live on the skin of their hosts, feeding on their blood, hair, or feathers (Price 1980; Schmid-Hempel 2011). External and internal parasites may have different effects on their host. They may, for instance, activate different parts of the immune system and/or drain the host out of different nutrients and energy (Schmid-Hempel 2011). Experimentally manipulating either ecto- or endoparasite levels in wild animals has been shown to affect reproductive success (Hudson 1986; Møller 1990, 1993; de Lope et al. 1998; Stien et al. 2002; Bustnes et al. 2006), chick survival (Newborn and Foster 2002; Amundson and Arnold 2010), territorial aggressiveness (Fox and Hudson 2001), and adult survival (Slattery and Alisauskas 2002; Hanssen et al. 2003). The present study aimed to experimentally manipulate both ecto-and endoparasite levels independently. While several studies manipulating parasite loads in wild animals (see above) have measured reproductive and other fitness-related parameters in wildlife, there are fewer reports on physiological variables, such as immune function and oxidative status (but see Mougeot et al. 2010). These variables are important because oxidative status and immune function are linked (Costantini and Møller 2009), and oxidative stress may be one of the important proximate factors that constrain fitness-related traits, such as reproduction and survival (Beckman and Ames 1998).

Endoparasites often use top predators as definitive hosts (Crompton and Nickol 1985). Birds of prey are therefore commonly infected with a variety of endoparasites, including nematodes, trematodes, cestodes, acanthocephalans, and coccidians (Rausch 1983; Upton et al. 1990; Cawthorn 1993; Smith 1993). Additionally, birds of prey often build large nests that are used for several consecutive years, thus enabling ectoparasites, such as fleas and lice, to winter in the nests and readily infest birds when breeding commences in spring (reviewed by Philips and Dindal 1977).

**Figure 1:**
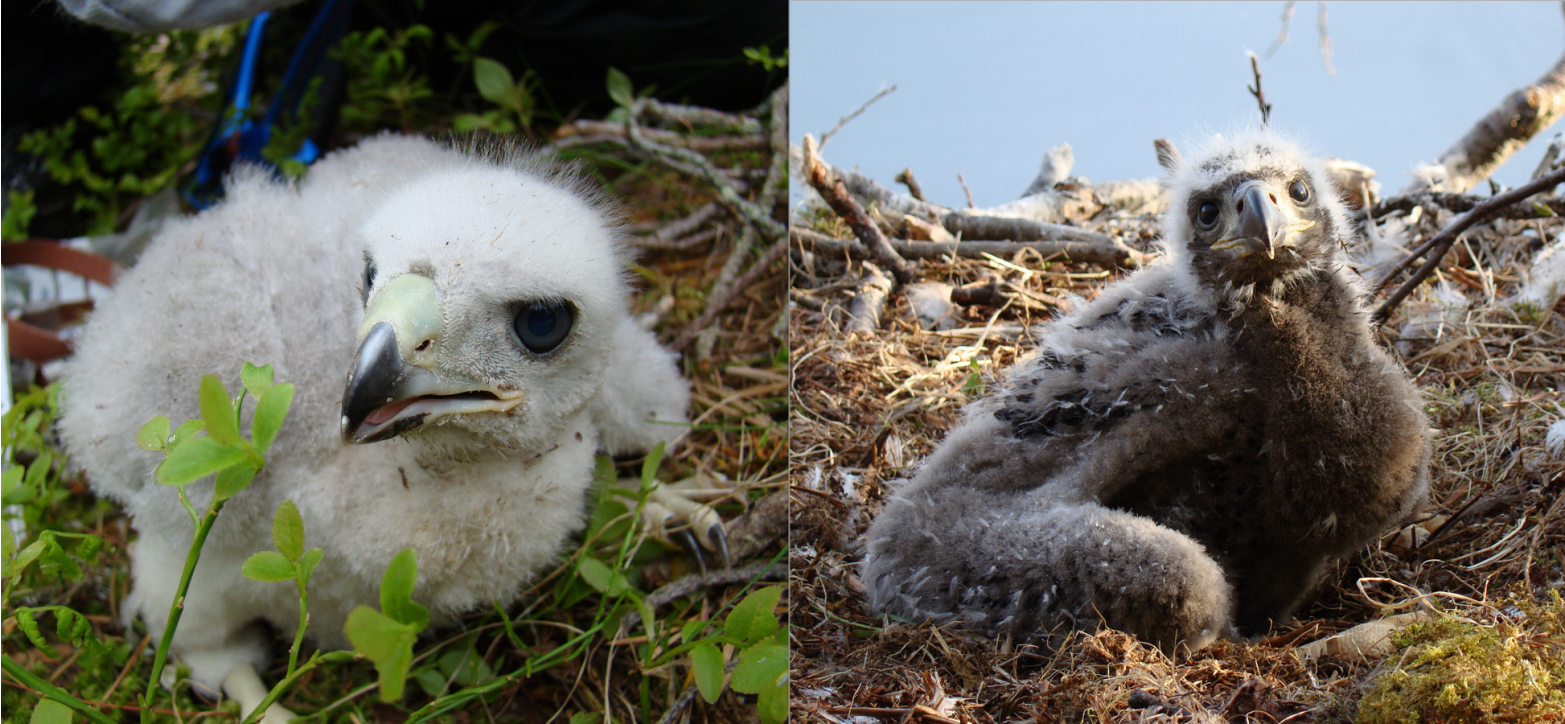
Northern goshawk *Accipiter gentilis* (left) and white-tailed sea eagle *Haliaeetus albicilla* (right) nestlings. Photo: Manuel Ballesteros.

The objective of this study was to unravel possible links between parasitism, immune function, and oxidative status by relieving chicks of two different raptor species (northern goshawk *Accipiter gentilis* and white-tailed sea eagle *Haliaeetus albicilla*) from two different groups of parasites: endo- and ectoparasites (Fig. 1). First, the study species represent different northern ecosystem food chains, northern goshawks residing in woodlands, and white-tailed sea eagles in coastal areas. As northern goshawks feed mainly on terrestrial birds and small mammals, whereas white-tailed sea eagles mainly feed on marine prey, there are likely qualitative and quantitative differences in endoparasites between both species. Also, while northern goshawks show a high degree of sexual size dimorphism, males' body mass averaging only 61% of females' body mass (mean adult male 865g; female 1414 g), white-tailed sea eagles are rather less dimorphic, males' body mass averaging 72% of females' body mass (adult males 4014 g; adult females 5572 g) (Cramp and Simmons 1980). Conducting the same experiment in two species differing in ecology and sex-specific characteristics should give us insight into the generality of mechanisms and evolutionary adaptations regulating the balance between infectious organisms, immunity, and oxidative status.

We randomly assigned nests and chicks to two different parasite-reducing treatments and two different control treatments. In early spring, nests were either sprayed with pyrethrin to reduce levels of ectoparasites or left unsprayed as control nests. Within each nest, we randomly treated chicks orally with either an antihelminthic medication (fenbendazole) to reduce endoparasites or sterile water as control treatment. Chicks were thus placed in four treatment groups: (1) control (ectocontrol, endocontrol), (2) ectoparasite treatment (anti-ecto, endocontrol), (3) endoparasite treatment (ectocontrol, anti-endo), and (4) both ectoparasite and endoparasite treatment (anti-ecto, anti-endo). We investigated the treatment effects on plasma (1) total antioxidant capacity (TAC; an index of nonenzymatic circulating antioxidants), (2) total oxidant status (TOS; a measure of plasma oxidants), and (3) immunoglobulin levels (a measure of humoral immune function). We predicted that treatment against ecto- and endoparasites would reduce the plasma levels of immunoglobulins, as alleviating levels of infectious organisms should ease up the antibody response. As immune responses are costly, both directly in terms of energy and nutrients and indirectly as they may increase oxidative stress (OS), we expected that antiparasite treatments would reduce oxidant levels and increase antioxidant levels. Furthermore, we expected birds treated against both endo- and ectoparasites to have the lowest immunoglobulin and oxidant levels, indicating better health than the other treatment groups.

## Materials and Methods

### Ethics, study design, and sampling

The study was conducted in Troms County, Northern Norway, on chicks of two predatory bird species: white-tailed sea eagle and northern goshawk. This study was approved by the National Animal Research Authority of Norway. From mid-February to mid-March, prior to the breeding seasons of 2008 and 2009, all accessible known territories and nests of northern goshawks and white-tailed sea eagles were visited. Some of these nests were randomly chosen to be treated with the commercially available ectoparasite removal spray SprayMax (Borregaard Industries Limited, active ingredient pyrethrin). These nests were treated for about 1 min, while control nests received a visit of similar length as treated nests. The treatment of the nests was performed 2–3 months before egg laying and thus targeted wintering stages of nest parasites to reduce parasite intensities, although one cannot exclude that adult birds occupying treated nests might have brought new parasites. Territories were thereafter visited during May to check nest occupancy. The occupied nests were again visited shortly after hatching in June. Northern goshawks occupied 7 nests in 2008 and 9 in 2009, and white-tailed sea eagles occupied 4 and 9 nests in 2008 and 2009, respectively. Northern goshawk nests contained 1–4 chicks (2.06 ± 0.05), and white-tailed sea eagle nests contained 1–2 chicks (1.31 ± 0.04). During this visit, chicks were randomly treated orally with either the antihelminthic Panacur® (active ingredient fenbendazole) (1 mL for northern goshawk chicks and 2 mL for white-tailed sea eagle chicks) or a corresponding amount of sterile water for control birds, see Hanssen et al. (2003) and Bustnes et al. (2006) for more details on this treatment in wild birds. Consequently, each chick received a combination of two different experimental treatments in this 2 × 2 crossed factorial design (Table 1). In order to ensure that each chick was randomly assigned to experimental groups, we tested initial size-corrected body mass, tail length, and tarsus length in relation to the experimental groups (all *P* > 0.26). Nests were visited again later (white-tailed sea eagles 18.5 ± 1.8 days, northern goshawks 13.4 ± 0.7 days) in order to obtain a blood sample for analysis of plasma immunoglobulins and oxidative status. The blood was sampled from the brachial vein (0.1–4.0 mL; heparin-coated syringe) and centrifuged on the same day at 1500 G for 10 min, and 1 mL supernatant plasma was transferred to a sterile 1.5 mL Eppendorf® tube and frozen at −20°C until analysis. On both visits, all chicks were weighed and their tail feathers were measured in order to register mass gain and growth. In order to minimize the time spent at the nest and thus the invasiveness of the study, we did not attempt to quantify the reduction in parasite levels in relation to the treatment. Yet, several studies have shown that fenbendazole is effective against various intestinal parasites in birds, for example nematodes, lungworms, and cestodes (Norton et al. 1991; Yazwinski et al. 1992, 1993). Lawrence (1983) showed that a single treatment with fenbendazole eliminated all nematode parasites in 221 of 230 birds from 38 species of six orders. Furthermore, treatment of nests with pyrethrin has been shown to reduce levels of ticks and fleas on nestlings (Szep and Møller 1999; Fessl et al. 2006) and nests (Dufva and Allander 1996; Christe et al. 2000, 2002). Further details on sampling can be found in Sonne et al. (2010).

**Table 1:** Sample sizes (number of chicks) for the double parasite removal experiment in (A) northern goshawk and (B) white-tailed sea eagle. Nests were treated with either an insecticide (pyrethrin) or left as controls, number of nests treated or controls are presented in brackets. After hatching, chicks were treated with either an anti-endoparasite treatment (fenbendazole) or control (sterile water)

Treatments	Control (10 nests)	Anti-ectoparasite (6 nests)	Total (16 nests)
(A) *Northern goshawk*			
Control	**11**	**7**	18
Anti-endoparasite	**10**	**5**	15
Total	21	12	**33**

Treatments	Control (6 nests)	Anti-ectoparasite (7 nests)	Total (13 nests)
(B) *White-tailed sea eagle*			
Control	**4**	**4**	8
Anti-endoparasite	**3**	**6**	9
Total	7	10	**17**

### Sexing

DNA was extracted from feather samples (approx. 2 mm root tip) or nestling blood (5–10 *μ*L) using Nexttec™ Genomic DNA Isolation Kit for Tissue and Cells. We used primers 2550F and 2718R (Fridolfsson and Ellegren 1999). PCR amplifications were performed in 25 *μ*L reaction volumes using approximately 10 ng of genomic DNA, and final concentrations of 0.2 *μ*M each primer, 0.2 mM each dNTP, 2.5 mM MgCl2, and 0.04U *μ*L^−1^ of TaKaRa LA Taq™. We typically used the following PCR profile on a Mastercycler® ep gradient S instrument: initial denaturation at 94°C for 2 min, then 7 cycles of denaturation at 94°C for 20 s, touchdown annealing at 60–54°C (minus 1°C per cycle) for 30 s, and extension at 72°C for 40 s, followed by 30 cycles with constant annealing temperature at 54°C, and a final incubation at 72°C for 1 min. PCR products were run on 1.5% agarose gels and visualized using SYBR® Safe. Male white-tailed sea eagles produced one band (approx. 650 bp), while female eagles produced two bands (approx. 650 and 450 bp), corresponding to their ZZ and ZW genotypes, respectively. In northern goshawks, due to differential amplification efficiency of the Z- and W-specific gene copies (Fridolfsson and Ellegren 1999), males produced the Z-specific band as in the white-tailed sea eagles (approx. 650 bp), while amplification of female samples produced the W-specific band only (approx. 450 bp).

### Immunoglobulins

Immunoglobulins are the most important serum proteins involved in humoral immune response in birds (Roitt et al. 1998). Plasma immunoglobulin Y (IgY), which is the avian equivalent to mammalian IgG, was assessed using a sensitive enzyme-linked immune absorbent assay (ELISA) method. Commercial antibodies were used as reported by Martinez et al. (2003). We adapted this method for raptor chicks by determining the appropriate plasma dilution (1/16,000). IgY levels were expressed in units of absorbance (Victor3 multilabel plate reader, Perkin Elmer, Turku, Finland). This analysis required 2 *μ*l of plasma.

### Oxidative status indices

Plasma total antioxidant capacity (TAC) was measured using a commercial kit (TAC assay kit, RL0017, Rel Assay Diagnostics, Gaziantep, Turkey) and an automated biochemical analyzer (Cobas c 111 analyzer, Roche Diagnostics, Rotkreuz, Switzerland). TAC assesses the nonenzymatic antioxidants (of both dietary and endogenous sources) present in the plasma sample. Antioxidants in the sample reduce dark blue-green-colored ABTS radical to colorless reduced ABTS form. The change of absorbance at 660 nm is related with total antioxidant level of the sample. The reaction rate is calibrated with Trolox, a widely used standard for TAC measurement assays, and the assay results are expressed in mmol Trolox equivalent L^−1^ in reference to a standard curve.

Plasma total oxidant status (TOS) was measured using a commercial kit (TOS assay kit, RL0024, Rel Assay Diagnostics) and an automated biochemical analyzer (Cobas c 111 analyzer, Roche Diagnostics). TOS assesses both hydrogen peroxide components (that are able to break down to produce reactive pro-oxidants and can also be used as an indicator of the superoxide dismutase activity) and lipid hydroperoxides (which indicate damage to lipids) (Erel 2005). Oxidants present in the sample oxidize the ferrous ion–chelator complex to ferric ion. The oxidation reaction is prolonged by enhancer molecules, which are abundantly present in the reaction medium. The ferric ion makes a colored complex with chromogen in an acidic medium. The color intensity, which can be measured spectrophotometrically, is related to the total amount of oxidant molecules present in the sample. The assay is calibrated with hydrogen peroxide (H_2_O_2_), and the results are expressed in *μ*mol H_2_O_2_ equivalent L^−1^ in reference to a standard curve. This analysis required 21 *μ*l of plasma. Oxidative status assays were performed in December 2009. All the samples have been run in the same conditions with the same commercial kits and a biochemical analyzer which guarantees a high repeatability: Intra-assay variation was 0.60% for TAC (*N* = 10) and 2.72% for TOS (*N* = 10). For more information on the specific analyses performed see Bourgeon et al. (2012).

### Experimental design and statistical methods

Sample sizes may differ slightly between analyses because not all laboratory tests could be run on all samples. We therefore present the sample size used for each analysis in Table 2. Each response variable was analyzed in a mixed analysis design (proc mixed in SAS 9.3). Nest identity was always included as a random variable to avoid pseudoreplication of chicks within nests. Of the 13 territories (nest sites) used during the two years for white-tailed sea eagles, one territory was included in the study in both years, and the corresponding numbers for goshawk were 16 territories in total of which 2 were used in two years. Because each territory may differ in quality, we also included territory identity as a random variable in all the analyses. We always started with a full model with nest and territory as random variable and the two experimental treatments and their interaction as fixed variables. In addition, we included species, year, sex, and their interactions with the two experimental treatments as cofactors in the full model unless otherwise stated. Chick body mass at the last capture was also used as covariate in the full models. We also introduced the three-way interaction between species and experimental groups to the initial model, in order to assess whether there were differences in how the two study species responded to the experiment. From this full model, we sequentially removed nonsignificant effects; we retained fixed variables that were nonsignificant if an interaction effect containing this variable was significant. We only present the results of the final models with covariates/cofactors (where present) below. Mean values are presented as mean ± standard error. All the analyses were performed with the statistical software SAS version 9.3.

**Table 2:** Effects of treating against ectoparasites (ecto) in nests and against endoparasites (endo) in chicks of northern goshawk (GH) and white-tailed sea eagle (WTSE) in Northern Norway on immunoglobulins, total oxidant status (TOS), and total antioxidant capacity (TAC). C, control group; T, treated group. All variables presented are from the resulting models of mixed model analyses with nest and territory as random variable with restricted maximum-likelihood estimation method. Estimates are presented for variables with *P*-values less than 0.05 and are least square means from the presented final models

Dependent variable	*n*	Main effects	*F*-value/*P*-value	Mean values (± std. err)	Covariates/ cofactors	*F*-Value/*P*-value	Mean values (± std. err)	Interaction effects	*F*-Value/*P*-value
Immunoglobulins	44	Anti-ectoparasite	*F*_1,12_ = 1.13/*P* = 0.31		Sex	*F*_1,12_ = 0.05/*P* = 0.82		ecto × sex (Fig 2A)	*F*_1,12_ = 7.08/***P*** **= 0.02**
		Anti-endoparasite	*F*_1,12_ = 7.05/***P*** **= 0.02**	C: −0.59 ± 0.04 T: −0.54 ± 0.04	Species	*F*_1,12_ = 46.5/***P*** **< 0.0001**	GH: −0.69 ± 0.02 WTSE: −0.54 ± 0.04	species × endo (Fig 2B)	*F*_1,12_ = 3.83/*P* = 0.07
TOS	50	Anti-ectoparasite	*F*_1,18_ = 0.17/*P* = 0.68		Body mass	*F*_1,18_ = 8.42/***P*** **= 0.01**	−0.00009 ± 0.00003^1^	endo × ecto (Fig 2C)	*F*_1,18_ = 10.77/***P*** **= 0.004**
		Anti-endoparasite	*F*_1,18_ = 0.14/*P* = 0.71						
TAC	50	Anti-ectoparasite	*F*_1,19_ = 6.637/***P*** **= 0.019**	C: 0.28 ± 0.02 T: 0.37 ± 0.02				endo × ecto (Fig 2D)	*F*_1,19_ = 6.25/***P*** **= 0.022**
		Anti-endoparasite	*F*_1,19_ = 0.85/*P* = 0.37						

1 Regression coefficient.

## Results

### Sex and growth

To evaluate if any of the treatments or combination of treatments affected mass gain or growth in nestlings, we calculated the daily mass change and daily growth of tail feathers. Neither of these variables were significantly affected by any of the experimental treatments or the interaction between treatments (all *P*-values >0.25).

The sex analyses showed that 15 goshawk chicks were females and 18 were males. The corresponding numbers for white-tailed sea eagles were 6 females and 11 males. As expected, there was marked size dimorphism between the sexes in goshawks. Females were heavier than males (body mass females 1101 ± 44 g, males 783 ± 41 g, *F* = 37.40, *P* < 0.0001). Body mass was not significantly different between the sexes in white-tailed sea eagles even though female chicks tended to be heavier (body mass females 4408 ± 269 g, males 4100 ± 199 g, *F* = 0.85, *P* = 0.37).

### Humoral immunity

**Figure 2:**
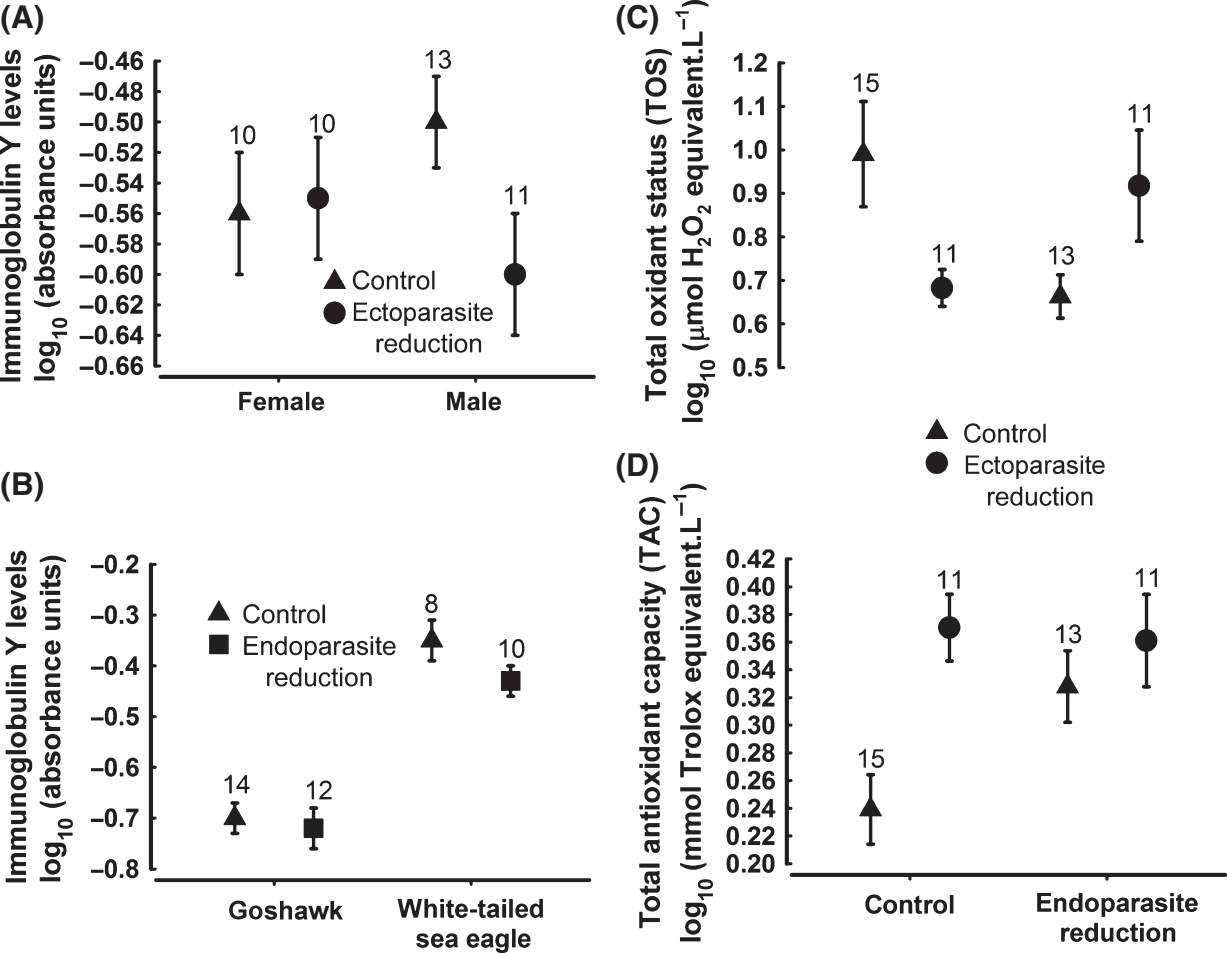
Effects of two different antiparasite treatments (against ectoparasites and endoparasites) on plasma concentrations of (A, B) immunoglobulin Y, (C) total oxidant status (TOS), and (D) total antioxidant capacity (TAC) in northern goshawk *Accipiter gentilis* and white-tailed sea eagle *Haliaeetus albicilla* nestlings. Values mean values ± SE.

Removing endoparasites led to a reduction in circulating immunoglobulin (Ig) plasma levels (Table 2). This difference was more pronounced in white-tailed sea eagle chicks (Table 2, Fig. 2B). Removing ectoparasites led to a reduction in Ig levels in males (Fig. 2A; Table 2). The level of immunoglobulins was also higher in white-tailed sea eagle chicks (Table 2).

### Oxidative status indices

TOS was higher in chicks receiving both treatments and birds not receiving any treatment (controls), when compared to receiving only one antiparasitic treatment (Fig. 2C; Table 2). TOS was also negatively related to body mass (Table 2).

TAC was lower in birds not receiving anti-ectoparasite treatment (Table 2). The significant interaction between treatments showed that the double control birds had significantly lower TAC levels when compared to birds receiving one or both treatments (Fig. 2D, Table 2).

## Discussion

The crossed experimental design of the present study allowed us to distinguish between the effects of treating against endo- and ectoparasites, and to explore the effects of treatment against both parasite systems in combination. White-tailed sea eagle chicks treated against internal parasites had reduced levels of immunoglobulins (Ig). We furthermore found that treating raptor nests with antiflea spray (pyrethrin) led to reduced Ig levels in male chicks. We also found that treatment against either ecto- or endoparasites reduced total oxidant status (TOS) in both northern goshawk and white-tailed sea eagle nestlings, while simultaneously administrating endo- and ectoparasite treatments did not lead to reduced TOS. All treatments against parasites led to higher total antioxidant capacity (TAC), when compared to controls.

We did not find any effects of the treatments on weight gain and tail feather growth in raptor chicks. Thus, it could not be concluded that parasites consume energy or resources that could be used for growth in chicks during the first month of life. However, the treatment effects on oxidative status and immune function may indicate that costs of parasitism are related to the physiology of growing individuals without affecting growth. It can, however, not be ruled out that the possible effects of antiparasite treatment on growth are manifested later in life than the 2–3 weeks after treatment where we measured potential effects.

Treating nests with antiflea spray (anti-ectoparasite treatment) led to a reduction in circulating immunoglobulins in male raptor chicks, and treatment against endoparasites also led to a reduction in circulating immunoglobulins in white-tailed sea eagle chicks. Thus, these treatments seemed to lead to a reduced investment in immune responses. Previous observational studies have documented a positive correlation between ectoparasitic infection and IgY levels (Moreno et al. 2008; King et al. 2011). Sex differences in T-cell-mediated immune function have been observed in bird chicks of other species (Fargallo et al. 2002; Müller et al. 2003; Tschirren et al. 2003) and may be related to, for example, differences in testosterone levels (Folstad and Karter 1992). However, higher testosterone levels may act as an immunosuppressor, and we would thus expect lower immunoglobulin levels in males, compared with females. In this study, the highest immunoglobulin levels were measured in male chicks of the control group, while females and treated males had lower levels. It has been suggested that in animals with sexual size dimorphism the largest sex may invest more in growth at the expense of, for example, immune function (Møller et al. 1998; Tschirren et al. 2003; Love et al. 2005). The observed sex differences may thus stem from the observed sexual size dimorphism where females are heavier than males in both species, although the size difference was not significant in white-tailed sea eagle chicks where sexual size dimorphism is not as marked as in goshawks. The smaller male chicks may invest more into immune defense, possibly explaining the high immunoglobulin levels in male control chicks. Treating nests with anti-ectoparasite medication thus seemed to enable male chicks to reduce their investment in immune defenses. Alternatively, the high Ig levels in male control chicks could mean that male chicks are more vulnerable to ectoparasitic infections, and the lower IgY levels in male chicks from treated nests may reflect lower infection levels. Interestingly, in the species with lower sexual size dimorphism (white-tailed sea eagles), treatment against endoparasites led to a reduction in Ig levels in both sexes.

In the present study, we found that TAC was lower in double control chicks. This may indicate that antioxidant levels are depleted in birds not treated against any parasites, perhaps as a consequence of higher immune activity related to defense against parasites, this is partly supported by the observed higher immunoglobulin levels in male chicks not treated against ectoparasites and white-tailed sea eagle chicks not treated against endoparasites. It should be noted here that there has been some controversy regarding the use of TAC as index of the overall activity of antioxidant system (i.e., Cohen and McGraw 2009), mainly because TAC may correlate strongly with uric acid (Costantini 2010) and therefore to a large part may reflect the activity of this antioxidant.

We furthermore found that control birds and birds receiving both anti-endo- and anti-ectoparasite treatments had higher TOS, while chicks receiving only one of the antiparasite treatments had reduced levels of oxidants. Reducing parasite levels appears to lead to a reduction in TOS, perhaps as a response to a concomitant reduction in ROS-producing immune responses. However, this does not explain why TOS was not reduced in chicks treated against both endo- and ectoparasites.

High TOS levels indicate high levels of oxidants in plasma, explanations for this may be related to depleted antioxidant levels, and this is, however, not probable here as TAC levels were high in this group. Therefore, it may be more plausible that activities increasing the level of oxidants are higher in this group.

Oxidant levels may increase when the immune system is active (Costantini 2008). However, an overactive immune system seems less likely for the birds that were treated against both endo- and ectoparasites. Neither could we find that these double-treated birds had higher levels of immunoglobulin which may have indicated high immune activity. However, the inflammatory response of the innate immune system generates high levels of ROS (Beckman and Ames 1998; Barja 2000), and this initial response is usually not reflected in immunoglobulin levels. Such an inflammatory response may have been triggered by the experimental removal of some types of parasites leading to higher infections with other types of macroparasites or microparasites such as bacteria and fungi (Van Oers et al. 2002; Pedersen and Antonovics 2013).

Oxidant levels may also increase as a consequence of increased metabolic rate (Wiersma et al. 2004; Halliwell and Gutteridge 2007; Costantini 2008). Interestingly, pharmacological treatment of Cape ground squirrels (*Xerus inauris*) with antiparasite drugs led to increased resting metabolic rate (RMR) (Scantlebury et al. 2007). The authors suggested that this increased RMR may be related to (1) suppression of RMR by parasitized hosts, (2) by-products from parasites that may reduce RMR (Richards and Edward 2000), or finally (3) that the antiparasite treatment may have stimulated the detoxification enzyme pathways (Swanepoel et al. 1999).

Another possibility is that the combined treatment did not lead to reduced OS levels because of a potential toxic side effect of the medications used. However, this does not seem very likely; firstly, treatment against ectoparasites was performed 2–4 months before hatching, so any toxic side effects of pyrethrin are unlikely; secondly, this substance has been used in numerous studies to remove ectoparasites in birds' nests without any reports of side effects (Møller 1990; Dufva and Allander 1996; Szep and Møller 1999; Christe et al. 2000, 2002). Furthermore, a putative side effect of fenbendazole used against endoparasites is unlikely as the group treated only with fenbendazole had reduced TOS. Also, fenbendazole doses 1000 times higher than the recommended dose were not reported to have any side effects (Newborn and Foster 2002).

The question should perhaps rather be; why did the double-treated chicks not show any reduction in TOS, as seen in the chicks only treated against one type of parasite? The results showed that it is challenging to distinguish between specific effects of treatment against endo- versus ectoparasites. For instance, each treatment administrated separately led to a reduction in TOS, but both treatments in combination increased TOS. Ectoparasitic treatment led to a reduction in immunoglobulin levels and increase in TAC, suggesting a reduced strain on the acquired immune system and increased antioxidant levels.

This experimental study documented complex but consistent relationships between antiparasite treatments and oxidative status in two raptor species. Future studies should explore these relationships in other species in order to further unravel the role of parasites in life-history traits of free-living organisms.
